# Appraisal and Evaluation of the Learning Environment Instruments of the Student Nurse: A Systematic Review Using COSMIN Methodology

**DOI:** 10.3390/healthcare11071043

**Published:** 2023-04-05

**Authors:** Marzia Lommi, Anna De Benedictis, Simona Ricci, Luca Guarente, Roberto Latina, Giuliana Covelli, Gianluca Pozzuoli, Maddalena De Maria, Dominique Giovanniello, Gennaro Rocco, Alessandro Stievano, Laura Sabatino, Ippolito Notarnicola, Raffaella Gualandi, Daniela Tartaglini, Dhurata Ivziku

**Affiliations:** 1 UOC Care to the Person, Local Health Authority Roma 2, 00159 Rome, Italy; 2 Clinical Direction, Fondazione Policlinico Universitario Campus Bio-Medico, 000128 Rome, Italy; 3 Department of Biomedicine and Prevention, University Tor Vergata, 00133 Rome, Italy; 4 Department of Health Promotion, Mother and Child Care, Internal Medicine and Medical Specialities, University of Palermo, 90127 Palermo, Italy; 5 Department of Traslational Medical Sciences, University of Campania “Luigi Vanvitelli”, 81100 Caserta, Italy; 6 Centre of Excellence for Nursing Scholarship, Order of Nurses of Rome, 00136 Rome, Italy; 7 Department of Clinical and Experimental Medicine, University of Messina, 98100 Messina, Italy; 8 Department of Health Professions, Fondazione Policlinico Universitario Campus Bio-Medico, 000128 Rome, Italy

**Keywords:** educational learning environment, clinical learning environment, COSMIN, psychometric propriety, systematic review, nursing students

## Abstract

Background: Nursing education consists of theory and practice, and student nurses’ perception of the learning environment, both educational and clinical, is one of the elements that determines the success or failure of their university study path. This study aimed to identify the currently available tools for measuring the clinical and educational learning environments of student nurses and to evaluate their measurement properties in order to provide solid evidence for researchers, educators, and clinical tutors to use in the selection of tools. Methods: We conducted a systematic review to evaluate the psychometric properties of self-reported learning environment tools in accordance with the Consensus-based Standards for the Selection of Health Measurement Instruments (COSMIN) Guidelines of 2018. The research was conducted on the following databases: PubMed, CINAHL, APA PsycInfo, and ERIC. Results: In the literature, 14 instruments were found that evaluate both the traditional and simulated clinical learning environments and the educational learning environments of student nurses. These tools can be ideally divided into first-generation tools developed from different learning theories and second-generation tools developed by mixing, reviewing, and integrating different already-validated tools. Conclusion: Not all the relevant psychometric properties of the instruments were evaluated, and the methodological approaches used were often doubtful or inadequate, thus threatening the instruments’ external validity. Further research is needed to complete the validation processes undertaken for both new and already developed instruments, using higher-quality methods and evaluating all psychometric properties.

## 1. Introduction

For decades, literature has been studying the correlation between student satisfaction and the learning environment because the students’ opinion is one of the elements to be taken into account to identify situations that promote or hinder learning and determine the success or failure of the course of study [1]. The learning environment is considered to be the social and organizational atmosphere in which interactions and communications between members of a learning group take place [2]. Learning environment, educational climate, and educational environment are used as synonymous concepts in literature [3,4,5,6,7,8]. The educational environment influences student behavior and has a strong effect on their results, satisfaction, and success [4]. Therefore, identifying the elements operating in the educational environment of a given path of study and evaluating their perception by students enables them to be modified to improve the learning experience in relation to teaching objectives [7]. Nursing education consists of theory and practice [8], therefore the learning environment includes both the educational and clinical aspects. The educational environment, in the strict sense, is considered a space, a physical structure (often identified as a classroom), where students develop knowledge, skills, attitudes, and professional values through lectures and case-study discussions [9]. On the other hand, the clinical environment is identified as the area in which nursing students apply knowledge and skills, integrating theory and practice while caring for patients. Learning environments that satisfy students enable them to achieve better and more promising learning outcomes [10]. The elements that contribute to making an optimal learning environment are: pedagogical atmosphere, teaching, relationships with educators, clinical tutors, nursing staff, educational equipment, and a physical environment [11,12,13]. Over the years, various tools have been developed to assess nursing students’ perceptions of their clinical learning experience. In fact, two reviews have been published in the literature that examined the clinical environment assessment tools published up until 2016 [14,15]. In the first review [14], conducted on the PubMed, CINAHL, and PROQUEST databases, the tools used to assess the clinical learning environment were identified and were available up until 2014. The second review [15], conducted on two databases (PubMed and CINAHL), with the Consensus-based Standards for the selection of health Measurement Instruments (COSMIN) guideline 2010 [16,17], evaluated the measurement properties of clinical environment assessment tools published up until 2016.

A systematic review of the tools to evaluate the educational sphere, which seems to be a fundamental part of the learning environment in the clinical sphere, has not been found in the literature.

Therefore, this study aimed (1) to identify the currently available tools for measuring the learning environments, both clinical and educational, of nursing students and (2) to evaluate their measurement properties in order to provide solid evidence for researchers, educators, and clinical tutors to use in the selection of tools.

## 2. Methods

### 2.1. Methodology and Search Strategy

We conducted a systematic review to evaluate the psychometric properties of self-reported learning environment measuring tools in accordance with the 2018 COSMIN Guidelines. The research was conducted on the following databases: PubMed, CINAHL, APA PsycInfo, and ERIC, until 13 February 2023. The search phases were conducted according to the PRISMA statement [18]. The search strategy used the search filters suggested by Terwee and colleagues [19], in addition to the key elements of the construct of interest (construct, population, and type of tools), combining them with the Boolean operators AND and NOT. Appendix A gives an example of the search strategy used on PubMed. EndNote version 8.2 [20] was used to manage the systematic review process. Development studies of tools that evaluated the educational or clinical learning environment and validation studies of tools already developed were included. The included articles were written in English and published in academic and peer-reviewed journals. Studies that did not have as their main objective evaluating the tools’ measuring properties of the learning environment (e.g., cross-sectional studies that measured only the Cronbach α) were excluded. We also excluded discussion and review protocols because this literature provides only limited information. Furthermore, articles that did not publish the tool within the article were excluded because, according to the COSMIN Guidelines, this was necessary for the evaluation of the tool by reviewers. The review protocol was published in the PROSPERO register (CDR42023408271)

### 2.2. Data Synthesis and Quality Assessment Tool

COSMIN guidelines were adopted during the data synthesis process. These guidelines were initially developed to conduct systematic reviews of Patient-Reported Outcome Measures (PROMs). In recent times, these have been adapted to healthy individuals or caregiver-reported outcome measures [21]. In accordance with the guideline, two reviewers independently evaluated the content validity of each instrument in three steps. First, the quality of the development study was evaluated with COSMIN Box 1, which examines the relevance of the new tool’s items and the comprehensiveness and comprehensibility of the pilot study or the cognitive interview. Second, the quality of the validation studies was evaluated with COSMIN Box 2, divided into 5 sections (from 2a to 2e), which examine relevance, comprehensiveness, and comprehensibility. Here, the reviewer group can choose which sections to complete (e.g., if the professional has not been consulted in the content validity study, sections 2d and 2e can be skipped). Third, all the evidence from the development and validation studies is summarized, then the reviewers evaluate the tool, and finally an overall score is determined based on relevance, comprehensiveness, comprehensibility, and content validity (from sufficient to indeterminate). Finally, confidence in the trustworthiness of the overall ratings (high, moderate, low, or very low) is determined using the modified Grading of Recommendations Assessment, Development, and Evaluation (GRADE) approach. The quality of the evidence is considered high when one or more studies present very good psychometric and confident results. The quality is moderate when imprecision or inconsistency is observed. The quality is low or very low when the level of confidence is limited or very small. According to the COSMIN 2018 guidelines, a level A rating is assigned when there is evidence for sufficient content validity and low-quality evidence for sufficient internal consistency. Level B is assigned when the scale cannot be classified as level A or C. Level C is assigned when high-quality evidence for an insufficient measurement property is present.

Subsequently, two reviewers independently evaluated the psychometric properties of the tools in a three-step process. First, the methodological quality of each study was assessed with the COSMIN Risk of Bias checklist. Secondly, each measurement property was evaluated according to the criteria of the measurement properties. Third, the evidence for each instrument was summarized with a rating on its psychometric properties (from sufficient to indeterminate) and quality of evidence (high, moderate, low, very low) using the GRADE approach.

In accordance with COSMIN guidelines, at the end of these procedures, recommendations can be made on the use of instruments consisting of: level A- recommended for use; level B- potentially recommended but requiring further study; and level C- not recommended for use.

To carry out evaluations on the validity of the contents and the psychometric properties, the review team used the Excel file downloadable from the COSMIN website.

### 2.3. Data Extraction

During the evaluation process, two researchers extracted data from studies, including instrument title, author, year, and country of publication of the study; type of study (development or validity study); definition of the measured concept; sample characteristics; the number of items; response system; and psychometric properties investigated.

## 3. Results

### 3.1. Results of the Studies Included in the Review

**Figure 1:**
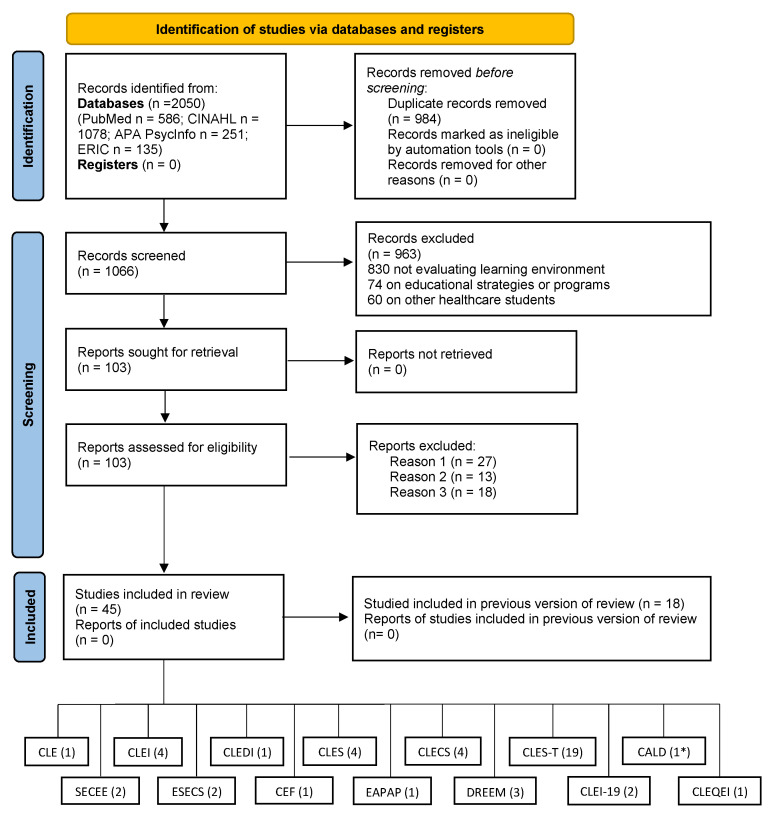
PRISMA 2020 flow diagram for new systematic reviews, which included searches of databases and registers only.

A total of 45 articles (11 development studies and 34 validation studies) containing 14 measurement tools were included in the review (see Figure 1). One of the articles included [22] is both a validation study (for the CLES-T) and a development study (for the CALD). These studies were conducted on different continents: Africa (Morocco: 1 study), Asia (China: 3 studies; Turkey: 3 studies; Hong Kong: 1 study; Iran: 1 study; Japan: 1 study; and Nepal: 1 study), Europe (Italy: 5 studies; Finland: 4 studies; Spain: 3 studies; Norway: 2 studies; Greece: 2 studies; Croatia: 2 studies; Austria: 1 study; Belgium: 1 study; Sweden: 1 study; Germany: 2 studies; Slovenia: 1 study; and Portugal: 1 study), Oceania (Australia: 5 studies and New Zealand: 1 study), and America (USA: 2 studies). The instruments assessed the clinical traditional learning environment (9 instruments: CLE, SECEE, CLES, CLES-T, CALD, CLEQEI, CLEI, CLEI-19, and CLEDI), the clinical traditional and simulated environment (2 instruments: ESECS and CLECS), the clinical placement environment (CEF), and the educational learning environment (2 instruments: EAPAP and DREEM). The descriptions of the studies and the instruments with their psychometric properties are presented in Table 1.

Note: Reason 1: instruments not included in article; Reason 2: not validation studies (e.g., survey); Reason 3: studies evaluating only one psychometric property (e.g., Cronbach Alpha); (*) Notice that the CALD instrument development study also includes a validation of the CLES-T, so it should not be summarized together with the other validation studies.

### 3.2. Methodological Quality, Overall Rating, and GRADE Quality of Evidence

In the evaluation of the quality of the evidence, 9 instruments were rated Moderate (CALD, CLECS, CLEI, CLEI-19, CLES, CLES-T, DREEM, ESECS, and SECEE), 3 Low (CEF, CLEDI, and CLEQEI) and 2 Very Low (CLE and EAPAP). This was determined by the quality and quantity of the validation and development studies reviewed. However, as indicated by the COSMIN guideline, studies that scored low or very low were not excluded from further evaluation. In addition, in the determination of relevance, comprehensiveness, and comprehensibility and, consequently, content validity, some biases in the study design resulted in low scores (most doubtful). The most frequent sources of bias were in the instrument development procedures (qualitative methodology for identifying relevant items; doubtful presence of a trained moderator or interviewer; no interview guidelines included in the article; the doubtful process of recording and transcribing participants’ responses; doubtful independence of the data coding process; doubtful reaching of data saturation); and in the pilot tests (not at the requisite level of relevance, comprehensiveness, or comprehensibility of items to respondents; insufficient number of people enrolled in the pilot test or expert panel). See Table 2.

### 3.3. Psychometric Properties, Overall Rating, and GRADE Quality of the Evidence

The next stage of evaluation focused on the psychometric properties of the instruments tested in the articles included in the review. They scored 5 instruments as high quality (CEF, CLEI-19, CLEQEI, EAPAP, and SECEE), 2 as Moderate (CLE and CLEDI), 4 instruments as Low (CALD, CLECS, CLES, and CLES-T), and 3 as Very Low (CLEI, DREEM, and ESECS). These ratings were determined by the procedures used to test psychometric properties and were affected by some biases. For example, low scores were given for structural validity if the sample size in the analysis was not adequate. Based on the psychometric properties investigated in the studies and reported in Table 1, we were able to assess whether they met the criteria for good measurement properties reported in the COSMIN guidelines. Finally, based on the quality of the studies and the psychometric properties of the instruments, we allocated recommendations according to the modified GRADE method indicated by the COSMIN guidelines.

### 3.4. Learning Environment Instruments

All the instruments included in the review were developed and validated to measure the nature of the learning environment, whether clinical or educational. We present here a brief narrative overview of the instruments. For a complete overview of the instruments and the procedures adopted in their development and validation, see Table 1.

The first tool developed to assess the clinical learning environment is the Clinical Learning Environment (CLE) tool. This instrument was developed based on the theories of Orton (1981) [66], who conducted a survey of the learning environment in hospital wards and generated a scale consisting of 124 items. Dunn and Burnett, with a panel of 12 experienced clinical educators, considered only 55 items valid and then, through factor analysis, confirmed an instrument consisting of 23 items and 5 subscales: staff-student relationships, nurse-manager commitment, patient relationships, interpersonal relationships, and student satisfaction. Only one instrument development study that met the inclusion criteria was identified by the review, and it was rated as “inadequate” for methodological quality because it was affected by the expert panel’s doubtful description of assessment procedures and the absence of a pilot test on nursing students [24]. The GRADE recommendation grade was C because of inconsistent content validity, very low methodological quality of studies, and insufficient internal consistency (Cronbach’s alpha being less than 0.70 in some factors of PCA and CFA).

The Dundee Ready Education Environment Measure (DREEM) was developed by Roff in 1997 to assess the educational environment of health professional trainees [67]. It originates from the results of a grounded theory study and subsequent panel of nearly 100 health educators from around the world, with subsequent validation by over 1000 students in countries as diverse as Scotland, Argentina, Bangladesh, and Ethiopia, to measure and diagnose educational environments in the health professions. It has been used internationally in different contexts, mainly with medical students, but also with other health professionals. The instrument consists of 50 items and 5 subscales: perception of learning, perception of teachers, social self-perception, perception of atmosphere, and academic self-perception. Three validation studies were included in the review, all of which reported sufficient content validity, moderate qualitative evidence (+/M), and sufficient though low internal consistency of the instrument (+/L), achieving a level A recommendation [58,59,60].

The Student Evaluation of Clinical Education Environment (SECEE) evaluates the clinical learning environment and was developed and validated by Sand-Jecklin in 1998 [64]. This instrument is based on the theoretical framework of cognitive apprenticeship, which states that students apply conceptual knowledge tools in a real-world environment while being guided by experienced professionals. Versions of the SECEE have evolved over time. Currently, the latest version is SECEE version 3, consisting of 32 items and 3 subscales: instructor facilitation, preceptor facilitation, and learning opportunities. Two validation studies were included in the review [65,68], and based on these, a grade of recommendation A was given for high quality of evidence, high internal consistency of the instrument, and sufficient content validity of moderate quality.

The Clinical Learning Environment Inventory (CLEI), which assesses the clinical learning environment, was developed and validated by Chan in 2001 [32,33,34]. It has been evaluated in four published journal articles, including three development articles and one validation article [32,33,34,35]. The instrument was developed based on the literature review and by modifying the College and University Classroom Environment Inventory (CUCEI) by Fraser and colleagues [69] (Assessment of Classroom Psychological Environment; Perth, Australia: Curtin University of Technology). Nearly 10 years later, Newton and colleagues (2010) modified 10 items from the “Actual” CLEI version, replacing the word “clinical teacher” with “preceptors,” and conducted a PCA for the first time [33]. The instrument contains 35 items and 5 subscales (each containing 7 items): individualization, innovation, involvement, personalization, and task orientation. The instrument has two formats: the “Actual” form, which measures the current clinical environment, and the “Preferred” form, which measures the preferred clinical environment. The instrument is not recommended for use (GRADE level C) because: studies showed moderate qualitative evidence, the instrument has inconsistent content validity (±/M), the internal consistency of the instrument is insufficient, and the quality of evidence of psychometric properties assessed is very low (-/VL).

In 2002, Saarikoski and Leino-Kilpi developed the Clinical Learning Environment and Supervision Instrument (CLES) [37]. The instrument originates from the theories of Quinn (1995), Wilson-Barnett et al. (1995), and Moss and Rowles (1997). From a review of literature focused on clinical learning environments and the supervisory relationship [31,32], the authors categorized and summarized those items that could reflect the construct, and these were then tested in a pilot study. Subsequently, the number and type of items were changed and revised by a group of experienced clinical teachers [37]. The final version of the CLES scale consists of 27 items and 5 subscales: ward atmosphere, leadership style of the ward manager, premises of nursing care on the ward, premises of learning on the ward, and supervisory relationship. The CLES instrument has been translated and validated in several countries: Belgium [39], Cyprus [47], and Italy [13,38], and used in international comparative validation studies (Finland and the United Kingdom) [39]. Four articles were included in the review: one development review [37] and three validation reviews [13,38,39]. The recommendation grade of the instrument is B since it requires further study due to low but sufficient evidence of its internal consistency (+/L) and moderate and inconsistent content validity (±/M).

In 2006, Hosoda [29] developed the Clinical Learning Environment Diagnostic Inventory (CLEDI) based on Kolb’s 1984 theory of experiential learning, which emphasizes that the learning process occurs only after the student is able to integrate concrete emotional experiences with cognitive processes [70]. The CLEDI is an instrument that contains 35 items and has 5 subscales: affective CLE, perceptual CLE, symbolic CLE, behavioral CLE, and reflective CLE. Only Hosoda’s instrument development study was included in the review, but due to the lack of a pilot study assessing students’ face validity, comprehensiveness, and comprehensibility, it scored low and had inconsistent content validity, earning a grade C recommendation.

In 2008, Saarikoski and colleagues modified the original CLES by including a new subscale related to the role of the nurse teacher (NL or T) to emphasize and define the importance of the nurse teacher in the clinical setting. The new scale, titled Clinical Learning Environment, Supervision, and Nurse Teacher (CLES-T) Scale, was validated in the same year [40]. A total of 19 studies were included: 1 development review [40] and 18 validation studies [39,44,45,46,47,48,49,50,51,52,53,54,55,56,57,58,59]. CLES-T also received a grade of B recommendation, needing further study. This is due to some less recent studies with some methodological and measurement property biases that contributed to degrees of low but sufficient evidence of internal consistency of the instrument (+/L) but moderate and inconsistent content validity (±/M).

In 2011, Salamonson and colleagues modified the CLEI, reducing the items from 35 to 19. The CLEI-19 is used to assess two generic domains common to clinical learning environments: clinical facilitator support of learning and satisfaction with clinical placement. In this review, we included two studies: one development study [34] and one validation study [35]. The instrument received a grade B recommendation, given the high quality of the evidence and sufficient assessment of the internal consistency of the instrument (+/H) and inconsistent content validity of moderate quality (±/M) due to the absence of pilot testing procedures and content and face validity by a panel expert.

In 2011, Porter and colleagues [23] developed an instrument to assess the support received by students during clinical internships with the overall goal of improving the quality of the students’ clinical experience. The Clinical Evaluation Form (CEF) consists of 21 items and 5 subscales: orientation, clinical educator/teacher, ward staff/preceptor and ward environment, final assessment/clinical hurdles, and university. Only the internal consistency of this instrument was assessed, receiving a score of sufficient and high quality. However, other important psychometric properties were not evaluated. In addition, the stage of item validation (e.g., whether it was undertaken by two researchers independently) and whether the items had been evaluated for relevance, comprehensiveness, and comprehensibility by nursing students were not clearly described. Therefore, the instrument was given a level B recommendation, requiring further study.

In 2014, Baptista and colleagues [62] developed an instrument to assess nursing students’ perceptions and satisfaction during simulated clinical experiences. The Escala de Satisfação com as Experiências Clínicas Simuladas (ESECS) was developed based on the results of a literature review and a phenomenological study describing students’ experiences in high-fidelity simulated practice using manikins. These studies resulted in a list of 17 items and 3 subscales: practical dimension, realism dimension, and cognitive dimension. Two studies were included in the review: one on development [62] and the other on validation [63]. The studies demonstrate moderate and sufficient content validity (+/M), but insufficient internal consistency with evidence quality rated as low, and therefore the instrument achieved a level B recommendation, needing further psychometric studies.

The Clinical Learning Environment Comparison Survey (CLECS) was developed by Leighton in 2015 [25] through a literature review, the results of which were evaluated and used by a panel of 12 academics with experience in simulation with manikins and clinical environments to generate the items and subscales. This instrument was used in two pilot studies to assess clarity. The final instrument consists of 27 items and 6 subscales: communication, nursing process, holism, critical thinking, self-efficacy, and teaching-learning dyad. Four studies were included in this review: one development [63] and three of validation [66,67,68]. The content validity of the instrument was inconsistent and moderate (±/M); this was due to the unclear description of procedures on students’ assessments of the comprehensiveness and comprehensibility of the instrument. However, the internal consistency of the instrument attained the level of sufficient, while the quality of the evidence was rated as low, and therefore the recommendation level of the instrument was B.

One of the studies on CLES-T documented the development of a new instrument, the Cultural and Linguistic Diversity (CALD) scale, that assesses the clinical learning environment. The theoretical framework for the development of the CALD originates from two systematic reviews conducted by Mikkonen and colleagues [22]. From the synthesis of data from the two reviews, following Thomas and Harden’s 3-step analysis process, 101 descriptive themes emerged that were compared with each item on the original CLES+T scale. Those that did not have corresponding items in the CLES+T scale were operationalized into measurable items to be used in the development of CALD. The final scale includes 21 items and 4 subscales: orientation into clinical placement, role of student, cultural diversity in the clinical learning environment, and linguistic diversity in the clinical learning environment. On the basis of methodological quality and results of psychometric properties, Mokkinen’s study was one of the best studies conducted, and therefore, even though only one instrument development study that met the inclusion criteria was included in the review, a level A recommendation was given.

The Clinical Learning Environment Quality Evaluation Index (CLEQEI) is an instrument developed in Italy by a group of researchers at the University of Udine in order to assess students’ perceived quality of clinical learning [36]. It is composed of 22 items investigating the quality of tutoring strategies, learning opportunity, safety and quality of care, self-learning, and the quality of the learning environment. It is the subject of one of the studies included in this review, which investigated several psychometric properties of the CLEQEI with good results, although the methodology for developing the instrument for assessing relevance, comprehensiveness, and comprehensibility was described unclearly and overly briefly. Only this one developmental study was included in the review, and the recommendation achieved was level B.

The Escala de Apoyo Académico en el Prácticum in Spanish (EAPAP) was developed by Arribas-Marìn in 2017 for the purpose of assessing students’ perceptions of academic support during internship [61]. The EAPAP consists of 23 items and 4 subscales: peer support, academic institution support, preceptor support, and clinical facilitator support. This study demonstrated inconsistent content validity with really low qualitative evidence (±/VL) but sufficient internal consistency with high methodological quality, and therefore, although there is only one study of the instrument development, it can be recommended at level B but needs further psychometric validation studies to be strongly recommended.

As highlighted in the results, these instruments are not all comparable with each other because, although they all assess the learning environment of nursing students, they focus on measuring specific aspects such as the traditional clinical learning environment (9 instruments: CLE, SECEE, CLES, CLES-T, CALD, CLEQEI, CLEI, CLEI-19, and CLEDI), the clinical traditional and simulated environment (2 instruments: ESECS and CLECS), the clinical placement environment (1 instrument: CEF), and the educational learning environment (2 instruments: EAPAP and DREEM).

To make the results of this review even more comprehensive, we conducted a qualitative analysis of the items belonging to all identified instruments to identify common and uncommon categories investigated by each instrument (see Table 3). Twenty-three categories were identified. Among the most common categories, “Quality of tutoring strategies” was explored by 11 instruments, followed by “Learning opportunities”, which was explored by 9 instruments including DREEM. “Quality of relationship with tutors”, “Quality of clinical learning environment”, and “Safety and quality of care” were each explored by 8 instruments. The most notable differences are found in the categories exploring “Self-efficacy in theoretical learning,” “Quality of relationship with tutors,” and “Quality of teaching strategies,” which are each explored by only two instruments: the DREEM and the EAPAP.

## 4. Discussion

In our systematic review, a total of 45 studies emerged that estimated the reliability and validity of 14 instruments in 22 different countries belonging to 5 continents. Most were conducted in Europe (24 studies). The first validation study was the CLE scale, and the last one was the CLEQEI in 2017 [36]. This indicates that this field of research spans more than 30 years, during which a tremendous amount of change has occurred in nursing programs, internship environments, and student profiles [71]. We can ideally divide the instruments based on their development into first- and second-generation instruments, in agreement with Mansutti and colleagues [15]. In fact, first-generation instruments such as CLE scales, CLEDI, CLES, CLES-T, DREEM, and the SECEE originated from major theories of learning established mainly in the 1980s and 1990s, while second-generation instruments, on the other hand, started from instruments previously established in clinical settings (such as CALD and CLEI-19) or from validation by expert panels of findings that emerged from literature reviews (see CLECS). Development and validation studies of second-generation instruments also appear to be better described in the procedures adopted, thus offering a better evaluation of evidence on methodological quality. In addition, in recent years, a trend has emerged to evaluate the validity and reliability of established instruments in different countries (e.g., the CLES-T), gather evidence on instrument validity, and compare data. The instruments that emerged consisted of from two (CLEI-19) to six (CLECS) factors or subscales and from 19 (CLEI-19) to 50 (DREEM) items.

Comparing results between different studies that used the same instruments was not always easy for several reasons. First, because the methodological quality adopted was heterogeneous. Second, because the validation studies were conducted at different times and some analyses may not have been known at the time or may have become obsolete over time. Other common problems encountered were that few studies estimated reliability. Although test-retest procedures should be easy to perform in an academic setting given the availability of students, the possibility that the duration and frequency of clinical rotations might have made it impossible to perform a second assessment for the same person should be considered. Internal consistency and structural validity were estimated for most of the instruments, but with methodological approaches of different quality, also compromising the quality of the results. Finally, convergent and criterion validity were assessed on a few occasions, especially in the first-generation instruments, due to the lack of available field knowledge and instruments that could be the gold standard for comparison.

### Limitations

One of the limitations of this review may have been that it included only peer-reviewed studies in English and Italian. Therefore, this may have resulted in a potential publication selection bias because other instruments may have been developed and diffused as gray literature or in different languages. The evaluation of the studies was based on the 2018 COSMIN guidelines, and some criteria required for the “very good” or “adequate” rating may not have been considered by authors of older studies, and this may have influenced the final evaluation of the instruments. Finally, it was not possible to assess the responsiveness of the instruments, that is, the ability of an instrument to detect change in the measured construct over time (as required by the COSMIN procedure), due to the absence of longitudinal studies among those included.

## 5. Conclusions

Fourteen tools that assess the quality of learning environments, both clinical and educational, have gone through a validation process so far. First-generation instruments have been developed from different learning theories, while second-generation instruments have been developed from the first generation by mixing, revising, and integrating several already-validated instruments. Not all relevant psychometric properties have been evaluated for the instruments, and often the methodological approaches used are doubtful or inadequate. In addition, a lack of homogeneity in the procedures for both assessing instrument relevance, comprehensiveness, and comprehensibility and for assessing psychometric properties emerged, thus threatening the external validity of the instruments. Future research must complete the validation processes undertaken for newly developed instruments and those already developed, but using higher-quality methods and estimating all psychometric properties.

## Figures and Tables

**Table 1:** Studies included in the review and psychometric properties of the instruments evaluated.

Tools	Author/ Year Publication/Country/Type of Study/Concept Evaluated	Sample	No.of Items/Subscale/Response System	Structural Validity	Internal Consistency	Other Psychometric Properties
**CALD**	Mikkonen et al., 2017 [22] Finland Development study Clinical learning environment	329 nursing students in 1st, 2nd, and 3rd-year courses	21 items 4 Subscales: orientation into clinical placement, role of student, cultural diversity in the clinical learning environment, and linguistic diversity in the clinical learning environment 5-point Likert (from 1 “fully disagree” to 5 “fully agree”)	EFA, 5 factors solution, 68% variance explained Content validity, a panel of 12 experts, CVI 0.75–1.00 Face validity, 10 nurse students	Total 0.88 Subscale: 0.77–0.85	Cross-cultural Validity (forward and backward translation) Hypothesis testing (convergent validity: CALD vs CLES-T): positive correlation between factor 1 CLES-T and Factor 3 CALD r = 0.62 *p* <0.01; positive correlation between Factor 2 CLES-T and Factor 4 CALD, r = 0.64 *p* < 0.01
**CEF**	Porter et al., 2011 [23] Australia Development study Clinical placement environment	178 nursing students in 1st and 2nd-year courses	21 items 5 subscales: orientation, clinical educator/teacher, ward staff/preceptor and ward environment, final assessment/clinical hurdles, and university 5-point Likert (from 1 “never” to 5 “always”)	Content and face validity, a panel of 3 experts (relevance, comprehensiveness, and comprehensibility) Face validity, 6 nurse students (comprehensiveness and comprehensibility)	Total 0.90 Subscales 0.73–0.91	
**CLE**	Dunn and Burnett, 1995 [24] Australia Development study Clinical Learning Environment	340 nursing students in 1st, 2nd, and 3rd-year courses	23 items 5 subscales: staff-student relationship, nurse management commitment, patient relationship, interpersonal relationship, and student satisfaction 5-point Likert (from 1 “strongly disagree” to 5 “strongly agree”)	PCA, orthogonal rotation, 4-factor solution, 34.6% explained variance CFA (testing scale with Orton’s theory): 5-factor solution GFI 0.86 AGFI 0.82 RMSR 0.07 Content validity (panel 12 members)	Subscales 0.60–0.83 (PCA) Subscales 0.63–0.85 (CFA)	
**CLECS**	Leighton, 2015 [25] USA Development study Clinical and simulated environment	422 nursing students from 4 colleges	27 items 6 subscales: communication, nursing process, holism, critical thinking, self-efficacy, and teaching-learning dyad 4-point Likert (from 1 “not meet” to 4 “well met”)	PCA, varimax rotation, 6 factors solution, 69.97% variance explained CFA, 6-factor solution (items 11 and 20 deleted), no index fit indicated	Total 0.94 Subscales 0.57–0.89 (traditional clinical environment) Total 0.90 Subscales 0.44–0.94 (simulated clinical environment)	Test-retest (recall period 2 week); r = 0.55, *p* < 0.05 (traditional environment); r = 0.58, *p* < 0.05 (simulated environment)
**CLECS**	Gu et al., 2018 [26] China Validation study Clinical and simulated environment	179 nursing students in 1st, 2nd, and 3rd-year courses	27 items 6 subscales: communication, nursing process, holism, critical thinking, self-efficacy, and teaching-learning dyad 5-point Likert (from 0 “not meet” to 4 “well met”)	PCA, varimax rotation, 5 factors solution, 61.43% variance explained (traditional environment) and 4-factor solution, 60.11% variance explained (simulated environment) CFA, 7-factor solution CFI 0.93 GFI 0.83 RMSEA 0.06 (traditional and simulated) Content validity, a panel of 4 experts Face validity, 10 student nurses	Total 0.75 Subscales 0.59–0.90 (traditional clinical environment) Total 0.95 Subscales 0.65–0.92 (simulated clinical environment)	Cross-cultural Validity (Forward-backward translation) Reliability: ICC: 0.63 consistency and 0.61 concordances (traditional clinical environment); and 0.93 consistency and 0.93 concordances (simulated clinical environment) Test-retest (recall period 2 weeks), r = 0.50 in a simulated and traditional environment
**CLECS**	Olaussen et al., 2020 [27] Norway Validation study Clinical and simulated environment	122 nursing students in 1st, 2nd, and 3rd-year courses	27 items of Simulated form the CLECS 6 subscales: communication, nursing process, holism, critical thinking, self-efficacy, and teaching-learning dyad 4-point Likert (from 1 “not applicable” to 4 “well met”)	CFA, 6-factor solution CFI 0.915 RMSEA 0.058 Content validity, a panel of 8 experts Face validity, 9 student nurses	Subscales 0.69–0.89	Cross-cultural Validity (guideline WHO 2018) Reliability: ICC: >0.50 (from 0.55 to 0.75)
**CLECS**	Riahi et al., 2022 [28] Iran Validation study Clinical and simulated environment	118 nursing students in 1st, 2nd, and 3rd-year courses	27 items of traditional form the CLECS 6 subscales: communication, nursing process, holism, critical thinking, self-efficacy, and teaching-learning dyad 5-point Likert (from 1 “not applicable” to 5 “well met”)	CFA, 6-factor solution CFI 0.829 RMSEA 0.078	Total 0.94 Subscales 0.82–0.94	Cross-cultural Validity (forward and backward translation) Hypotheses testing for construct validity (convergent validity) between the score of each item and the total score (from 0.809 to 0.976; *p* < 0.05) Hypotheses testing for construct validity (discriminant validity) between the score of each item and dimension (no good)
**CLEDI**	Hosoda Y., 2006 [29] Japan Development study Clinical learning environment	312 nursing students	21 items 5 factors: affective CLE, perceptual CLE, symbolic CLE, behavioral CLE, and reflective CLE 5-point Likert scale (from 1 “strongly disagree” to 5 “strongly agree”)	PCA, promax rotation, 5 factors solution, 52.45% variance explained Content validity, a panel of 22 experts (relevance, CVI)	Total 0.84 Subscales 0.65–0.77	Test-retest r = 0.76, *p* <0.01 Criterion validity (CLEDI and CLES), r = 0.76, *p* < 0.01 Hypotheses testing (known-groups technique: students and preceptors), *p* < 0.001
**CLEI**	Chan, 2001 [30], 2001 [31], 2002 [32]* Australia Development studies Clinical learning environment	108 nursing students in a 2nd-year course (quantitative phase) 21 nursing students (qualitative phase in Chan, 2001 [30])	Two forms: Actual CLEI and Preferred CLEI 35 items 5 subscales: individualization, innovation, involvement personalization, and task orientation 4-point Likert (from 1 “strongly agree” to 4 “strongly disagree”)		Subscales Actual form 0.73- 0.84 Subscales Preferred form 0.66- 0.80	Hypotheses testing (convergent validity): Actual forms with Preferred Form (r = 0.39−0.47)
**CLEI**	Newton et al., 2010 [33] Australia Validation study Clinical Learning Environment	513 nursing students in 2nd and 3rd-year courses	Actual CLEI form 42 items 6 subscales: personalization, student involvement, task orientation, innovation, satisfaction, and individualization 4-point Likert (from 1 “strongly agree” to 4 “strongly disagree”)	PCA, varimax rotation, 6 factors solution, 51% variance explained	Subscales 0.50–0.88	
**CLEI-19**	Salamonson et al., 2011 [34] Australia Validation study Clinical Learning Environment	231 nursing students in 1st, 2nd, and 3rd-year courses	19 items 2 subscales: clinical facilitator support of learning and satisfaction with clinical placement 5-point Likert (from 1 “strongly disagree” to 5 “strongly agree)	PCA, varimax rotation, 2-factor solution, 63.37% variance explained	Total 0.93 Subscales 0.92–0.94	Hypotheses testing (known-groups technique: work and non-working students) no-working students and clinical facilitator r = 0.037, *p* < 0.05; work students and satisfaction clinical placement, r = 0.038, *p* < 0.05
**CLEI-19**	Leone et al., 2022 [35] Italy Validation study Clinical Learning Environment	1095 nursing students in 1st, 2nd, and 3rd-year courses	19 items 2 subscales: clinical facilitator support of learning and satisfaction with clinical placement 5-point Likert (from 1 “strongly disagree” to 5 “strongly agree)	ESEM, 2-factor solution CFI 0.963 TLI 0.953 RMSEA 0.069 SRMR 0.037	Total 0–90 (alpha) Subscale 0.85–0.86 (Alpha) Total score 0.93 (Omega) Subscale 0.84- 0.89 (Omega)	
**CLEQEI**	Palese A. et al., 2017 [36] Italy Validation study Clinical Learning Environment	9606 nursing students in 1st, 2nd, and 3rd-year courses	22 items 5 subscales: quality of tutorial strategies, learning opportunities, safety and quality of care, self-learning, and quality of the learning environment 4-point Likert (from 0 “nothing” to 3 “very much”	EFA, 5-factor solution, 57,9% variance explained CFA, 5-factor solution CFI 0.966 TLI 0.960 RMSEA 0.050 SRMR 0.028 Content and face validity (experts and students)	Total 0.95 Subscales 0.82–0.93	Reliability: ICC (0.866 consistency and 0.864 concordance) Hyphothesis testing (discriminant validity) with CLES (r = 0.248, *p* < 0.0001) CLES-T (r = 0.733, *p* < 0.0001) Test-retest (recall period 2 weeks) 49.24 and 49.88
**CLES**	Saarikoski and Leino-Kilpi, 2002 [37] Finland Development study Clinical Learning Environment	416 nursing students in 2nd and 3rd-year courses	27 items 5 subscales: ward atmosphere, leadership style of the ward manager, premises of nursing care on the ward, premises of learning on the ward, and supervisory relationship 5-point-Likert (from 1 “fully disagree” to 5 “fully agree”)	EFA, 5-factor solution, 64% variance explained Face validity, a panel of 9 experts (comprehensiveness and comprehensibility)	Subscales 0.73–0.94	Hypothesis testing (convergent validity) of subscale CLES (correlation between “premises of nursing care” and “ward atmosphere”, r = 0.50 *p* < 0.005; between premises learning and premises nursing care, r = 0.46, *p* < 0.05)
**CLES**	Tomietto et al., 2009 [38] Italy Validation study Clinical Learning Environment	117 nursing students in 2nd and 3rd-year courses	27 items 5 subscales: ward atmosphere, leadership style of the ward manager, premises of nursing care on the ward, premises of learning on the ward, and supervisory relationship 5-point-Likert (from 1 “fully disagree” to 5 “fully agree”)		Total 0.96 Subscales 0.78–0.95	Cross-cultural Validity (forward and backward translation) Test-retest (recall period 3 weeks) r = 0.89
**CLES**	De Witte et al., 2011 [39] Belgium Validation study Clinical Learning Environment	768 nursing students of 1st, 2nd, and 3rd-year courses	27 items 5 subscales: ward atmosphere, leadership style of the ward manager, premises of nursing care on the ward, premises of learning on the ward, and supervisory relationship 5-point-Likert (from 1 “fully disagree” to 5 “fully agree”)	EFA, varimax rotation, 5-factor solution, 71,28% variance explained Content and face validity, a panel of 12 experts (relevance, comprehensiveness, and comprehensibility)	Total 0.97 Subscales 0.80–0.95	Cross-cultural Validity (forward and backward translation)
**CLES**	Burrai et al., 2012 [13] Italy Validation study Clinical Learning Environment	59 nursing students in 2nd-year courses	27 items 5 subscales: ward atmosphere, leadership style of the ward manager, premises of nursing care on the ward, premises of learning on the ward, and supervisory relationship 6-point-Likert (from 1 “fully disagree” to 6 “fully agree”)	PCA, promax rotation, 5-factor solution, 76.9% variance explained	Total 0.96 Subscales 0.81–0.96	
**CLES-T**	Saarikoski et al., 2008 [40] Finland Development study Clinical Learning Environment	965 nursing students in 1st, 2nd, and 3rd-year courses	34 items 5 subscales: supervisory relation, pedagogical atmosphere on the ward, role of nurse teacher, leadership style of the ward manager, and premises of nursing on the ward 5-point Likert (from 1 “fully disagree” to 5 “fully agree”)	EFA, varimax rotation, 5-factor solution; 67% variance explained	Total 0.90 Subscales 0.77–0.96	
**CLES-T**	Johansson et al., 2010 [41] Sweden Validation study Clinical Learning Environment	177 nursing students in 1st, 2nd, and 3rd-year courses	34 items 5 subscales: supervisory relation, pedagogical atmosphere on the ward, role of nurse teacher, leadership style of the ward manager, and premises of nursing on the ward 5-point Likert (from 1 “fully disagree” to 5 “fully agree”)	EFA, varimax rotation, 5-factor solutions; 60.2% variance explained	Total 0.95 Subscales 0.75–0.96	Cross-cultural Validity (forward and backward translation)
**CLES-T**	Henriksen et al., 2012 [42] Norway Validation study Clinical Learning Environment	407 nursing students in 1st, 2nd, and 3rd-year courses	34 items 5 subscales: supervisory relation, pedagogical atmosphere on the ward, role of nurse teacher, leadership style of the ward manager, and premises of nursing on the ward 5-point Likert (from 1 “fully disagree” to 5 “fully agree”)	PCA, varimax rotation, 5-factor solution; 64% variance explained	Total 0.95 Subscales 0.85–0.96	Cross-cultural Validity (forward and backward translation)
**CLES-T**	Tomietto et al., 2012 [43] Italy Validation study Clinical Learning Environment	855 nursing students in 1st, 2nd, and 3rd-year courses	34 items 5 subscales: supervisory relation, pedagogical atmosphere on the ward, role of nurse teacher, leadership style of the ward manager, and premises of nursing on the ward 5-point Likert (from 1 “fully disagree” to 5 “fully agree”)	EFA, oblimin rotation, 7-factor solution; 67.27% variance explained CFA, 7-factor solution CFI 0.929 RMSEA 0.061 SRMR 0.045 CFA, 5-factor solution CFI 0.817 RMSEA 0.097 SRMR 0.064	Total 0.95 Subscales 0.80–0.96	Cross-cultural Validity (forward and backward translation)
**CLES-T**	Bergjan et al., 2013 [44] Germany Validation study Clinical Learning Environment	178 nursing students in 1st, 2nd, and 3rd-year courses	34 items 5 subscales: supervisory relation, pedagogical atmosphere on the ward, role of nurse teacher, leadership style of the ward manager, and premises of nursing on the ward 5-point Likert (from 1 “fully disagree” to 5 “fully agree”)	EFA, oblimin rotation, 5-factor solution, 72.85% variance explained	Subscales 0.82–0.96	Cross-cultural Validity (forward and backward translation)
**CLES-T**	Watson et al., 2014 [45] New Zealand Validation study Clinical Learning Environment	416 nursing students in 1st, 2nd, and 3rd- year courses	34 items 5 subscales: supervisory relation, pedagogical atmosphere on the ward, role of nurse teacher, leadership style of the ward manager, and premises of nursing on the ward 5-point Likert (from 1 “fully disagree” to 5 “fully agree”)	EFA, 4-factor solution, 58.28% variance explained Face validity, a panel of 11 experts (relevance, comprehensiveness comprehensibility)	Subscales 0.82–0.93	
**CLES-T**	Vizcaya-Moreno et al., 2015 [46] Spain Validation study Clinical Learning Environment	370 nursing students of 1st, 2nd, and 3rd-year courses	34 items 5 subscales: supervisory relation, pedagogical atmosphere on the ward, role of nurse teacher, leadership style of the ward manager, and premises of nursing on the ward 5-point Likert (from 1 “fully disagree” to 5 “fully agree”)	EFA 5-factor solution, 66.4% variance explained CFA 5-factor solution CFI 0.92 GFI 0.83 RMSEA 0.065	Total 0.95 Subscales 0.80–0.97	Cross-cultural Validity (modify direct translation method)
**CLES-T**	Papastavrou et al., 2016 [47] Greece Validation study Clinical Learning Environment	463 nursing students of 1st, 2nd, and 3rd-year courses	34 items 5 subscales: supervisory relation, pedagogical atmosphere on the ward, role of nurse teacher, leadership style of the ward manager, and premises of nursing on the ward 5-point Likert (from 1 “fully disagree” to 5 “fully agree”)	EFA, varimax rotation, 5-factor solution, 67.4% variance explained Content validity, a panel of 5 experts (relevance, comprehensiveness comprehensibility)	Total 0.95 Subscales 0.81–0.96	Cross-cultural Validity (forward and backward translation)
**CLES-T**	Nepal et al., 2016 [48] Nepal Validation study Clinical Learning Environment	263 nursing students in 1st, 2nd, and 4th-year courses	34 items 5 subscales: supervisory relation, pedagogical atmosphere on the ward, role of nurse teacher, leadership style of the ward manager, and premises of nursing on the ward 5-point Likert (from 1 “fully disagree” to 5 “fully agree”)	EFA 5-factor solution, 85.7% variance explained	Total 0.93 Subscales 0.76–0.92	
**CLES-T**	Lovric et al., 2016 [49] Croatia Validation study Clinical Learning Environment	136 nursing students in 1st, 2nd, and 3rd-year courses	34 items 5 subscales: supervisory relation, pedagogical atmosphere on the ward, role of nurse teacher, leadership style of the ward manager, and premises of nursing on the ward 5-point Likert (from 1 “fully disagree” to 5 “fully agree”)	EFA 4-factor solution, 71.5% variance explained	Total 0.97 Subscales 0.77–0.96	Cross-cultural Validity (forward and backward translation) Test-retest: r = 0.55−0.79, *p* < 0.001
**CLES-T**	Mikkonen et al., 2017 [22] Finland Validation study Clinical Learning Environment	329 nursing students in 1st, 2nd, and 3rd- year courses	34 items 5 subscales: supervisory relation, pedagogical atmosphere on the ward, role of nurse teacher, leadership style of the ward manager, and premises of nursing on the ward 5-point Likert (from 1 “fully disagree” to 5 “fully agree”)	EFA, 8-factor solution, 78% variance explained	Total 0.88 Subscales 0.79–0.97	Hypothesis testing (convergent validity) with CLES-T (positive correlation between factor 1 CLES-T and Factor 3 CALD r = 0.62 *p* < 0.01; positive correlation between Factor 2 CLES-T and Factor 4 CALD, r = 0.64 *p* < 0.01)
**CLES-T**	Iyigun et al., 2018 [50] Turkey Validation study Clinical Learning Environment	190 nursing students in 3rd and 4th year courses	34 items 5 subscales: supervisory relation, pedagogical atmosphere on the ward, role of nurse teacher, leadership style of the ward manager, and premises of nursing on the ward 5-point Likert (from 1 “fully disagree” to 5 “fully agree”)	PCA, promax, 5-factor solution, 62% variance explained Content validity, a panel of 9 experts (relevance, comprehensiveness, and comprehensibility) CVI 0.96 Face validity, 10 nursing students (comprehensiveness and comprehensibility)	Subscales 0.76–0.93	Cross-cultural Validity (forward and backward translation) Hypothesis testing (convergent validity) with CLES (*p* < 0.05) Test-retest: r = 0.29−0.43, *p* < 0.005
**CLES-T**	Atay et al., 2018 [51] Turkey Validation study Clinical Learning Environment	602 nursing students in 1st, 2nd, and 3rd-year courses	34 items 5 subscales: supervisory relation, pedagogical atmosphere on the ward, role of nurse teacher, leadership style of the ward manager, and premises of nursing on the ward 5-point Likert (from 1 “fully disagree” to 5 “fully agree”)	EFA, 6-factor solution, 64% variance explained CFA (fit index not specified)	Total 0.95 Subscales 0.75–0.96	Cross-cultural Validity (forward and backward translation)
**CLES-T**	Zvanut et al., 2018 [52] Croatia Validation study Clinical Learning Environment	232 nursing students in 1st, 2nd, 3rd, and 5th-year courses	34 items 5 subscales: supervisory relation, pedagogical atmosphere on the ward, role of nurse teacher, leadership style of the ward manager, and premises of nursing on the ward 5-point Likert (from 1 “fully disagree” to 5 “fully agree”)	PCA, varimax rotation, 5-factor solution, 67.69% variance explained Face validity, 232 students (comprehensiveness and comprehensibility)	Total 0.96 Subscales 0.78–0.95	Cross-cultural Validity (forward and backward translation) Test-retest: (*p* < 0.05)
**CLES-T**	Mueller et al., 2018 [53] Austria Validation study Clinical Learning Environment	385 nursing students in 1st, 2nd, and 3rd-year courses	34 items 5 subscales: supervisory relation, pedagogical atmosphere on the ward, role of nurse teacher, leadership style of the ward manager, and premises of nursing on the ward 5-point Likert (from 1 “fully disagree” to 5 “fully agree”)	PCA, promax rotation, 4-factor solution, 73.3% variance explained	Total 0.95 Subscales 0.83–0.95	
**CLES-T**	Wong and Bressington, 2021 [54] Hong Kong Validation study Clinical Learning Environment	385 nursing students in 1st, 2nd, and 3rd-year courses	34 items 5 subscales: supervisory relation, pedagogical atmosphere on the ward, role of nurse teacher, leadership style of the ward manager, and premises of nursing on the ward 5-point Likert (from 1 “fully disagree” to 5 “fully agree”)	EFA, oblique rotation, 6-factor solution Content validity, a panel of 6 experts (relevance, comprehensiveness comprehensibility), CVI 0.93, range 0.83–1.0 Face validity, 15 nursing students (comprehensiveness and comprehensibility)	Total 0.94 Subscales 0.73–0.94	Test-Retest (recall period 2 weeks), ICC 0.85%, 95% CI
**CLES-T**	Zhao et al., 2021 [55] China Validation study Clinical Learning Environment	694 nursing students in 1st, 2nd, and 3rd-year courses	27 items 4 subscales: supervisory relationship, pedagogical atmosphere, leadership style of the ward manager, and premises of nursing on the ward 5-point Likert (from 1 “strongly disagree” to 5 “strongly agree”)	PCA, oblimin rotation, 3-factor solution, 60.01% variance explained CFA CFI 0.97 GFI 0.95 RMSEA 0.058 SRMR 0.04	Total 0.82 Subscales 0.70–0.79	
**CLES-T**	Ozbicakci et al., 2022 [56] Turkey Validation study Clinical Learning Environment	135 junior and senior nursing students	34 items 5 subscales: supervisory relation, pedagogical atmosphere on the ward, role of nurse teacher, leadership style of the ward manager, and premises of nursing on the ward 5-point Likert (from 1 “fully disagree” to 5 “fully agree”)	CFA, 5-factor solution GFI 0.68 RMSEA 0.092 Content validity, a panel of 3 experts (relevance, comprehensiveness and comprehensibility)) Face validity, 10 nursing students (comprehensiveness and comprehensibility)	Total 0.86 Subscales 0.48–0.94	
**CLES-T**	Guejdad et al., 2022 [57] Morocco Validation study Clinical Learning Environment	1550 nursing students in 1st, 2nd, and 3rd-year courses	34 items 5 subscales: supervisory relation, pedagogical atmosphere on the ward, role of nurse teacher, leadership style of the ward manager, and premises of nursing on the ward 5-point Likert (from 1 “fully disagree” to 5 “fully agree”)	EFA, promax rotation, 5-factor solution, 55% variance explained CFA, 5-factor solution GFI 0.946 CFI 0.961 RMSEA 0.035 Face validity, 28 nursing students (comprehensiveness and comprehensibility)	Total 0.93 Subscales 0.71–0.92	Cross-cultural Validity (forward and backward translation) Test-retest: ICC 0.84
**DREEM**	Wang et al., 2009 [58] China Validation study Educational environment	214 nursing students in 1st, 2nd, and 3rd-year courses	50 items 5 subscales: perception of learning, perception of teachers, social self-perception, perception of atmosphere, and academic self-perception 5-point Likert (from 0 “strongly disagree” to 4 “strongly agree)	PCA, oblimin, 5-factor solution, 52.19% variance explained	Total 0.95 Subscales 0.62–0.90	Cross-cultural Validity (forward and backward translation)
**DREEM**	Rotthoff et al., 2011 [59] Germany Validation study Educational environment	1119 nursing students in 1st, 2nd, and 3rd-year courses	50 items 5 subscales: perception of learning, perception of teachers, social self-perception, perception of atmosphere, and academic self-perception 5-point Likert (from 0 “strongly disagree” to 4 “strongly agree)	EFA, orthogonal rotation, 5-factor solution, 41.3% variance explained	Total 0.92 Subscales 0.57–0.84	Cross-cultural Validity (forward and backward translation) Hypothesis testing (known-groups technique: between students and number of semesters attended), perception of teaching is negative as the number of semesters attended increases, r = −0.18, *p* < 0.001
**DREEM**	Gosak et al., 2021 [60] Slovenia Validation study Educational environment	174 nursing students in 1st, 2nd, and 3rd-year courses	50 items 5 subscales: perception of learning, perception of teachers, social self-perception, perception of atmosphere, and academic self-perception 5-point Likert (from 0 “strongly disagree” to 4 “strongly agree)	Content validity, a panel of 6 experts, CVI 1.0 except for item n. 20	Total 0.95	Cross-cultural Validity (reverse translation technique)
**EAPAP**	Arribas-Marìn et al., 2017 [61] Spain Development study Educational environment	710 nursing students in 2nd-year courses	23 items 4 subscales: peer support, academic institution support, preceptor support, and clinical facilitator support 10-point Likert (from 1 “never” to 10 “always”)	PCA, promax rotation, 4 factors solution, 74.77% variance explained CFA, 4-factor solution CFI 0.960 RMSEA 0.051	Total 0.92 Subscales 0.88–0.96	
**ESECS**	Baptista et al., 2014 [62] Spain Development study Clinical and simulated environment	181 nursing students in 4th and 5th-year courses	17 items 3 Subscales: practical dimension, realism dimension, and cognitive dimension 5-point Likert (from 1 “unsatisfactory” to 5 “very satisfactory”)	PCA, orthogonal varimax rotation, 3-factor solution (practical dimension, realism dimension, and cognitive dimension)	Total 0.91 Subscales 0.73–0.89	
**ESECS**	Montejano Lozoya et al., 2019 [63] Portugal Validation study Clinical and simulated environment	174 student nurses in 2nd, 3rd, and 4th-year courses	17 items 3 Subscales: practical dimension, realism dimension, and cognitive dimension 5-point Likert (from 1 “unsatisfactory” to 5 “very satisfactory”)	PCA, varimax rotation, 4-factor solution, 66.6% variance explained CFA, 4-factor solution CFI 0.877 RMSEA 0.094 Face and content validity (panel of 8 experts, relevance, comprehensiveness, and comprehensibility) Face validity (53 nursing students, comprehensiveness, and comprehensibility)	Total 0.91	
**SECEE**	Sand-Jeclklin, 2009 [64] USA Validation study Clinical learning environment	2768 inventories of nursing sophomore, junior, and baccalaureate students	32 items 3 subscales: instructor facilitation, preceptor facilitation, and learning opportunities 5-point Likert (from 1 “strongly disagree” to 5 “strongly agree”)	EFA, 4-factor solution CFA, varimax rotation, 3-factor solution with 59% variance explained SRMR 0.037	Total 0.94 Subscales 0.82–0.94	Hypothesis testing according to student level (sophomore, junior, and senior) *p* = 0.05 seniors value more positively than sophomores
**SECEE**	Govina et al., 2016 [65] Greece Validation study Clinical learning environment	130 senior nursing students	32 items 3 subscales: instructor facilitation (IFL), preceptor facilitation (PFL), and learning opportunities (LO) 5-point Likert (from 1 “strongly disagree” to 5 “strongly agree”)	CFA, 3-factor solution CFI 0.92 RMSEA 0.052	Total 0.92 Subscales 0.84–0.89	Cross-cultural Validity (backward forward translation) Reliability (2 weeks di intervallo): ICC: 0.85–0.90, *p* < 0.0005 Hypothesis testing (discriminant validity) with CLES (highest between Ward atmosphere-PFL 0.537, and lowest between learning on the ward-IFL 0.163)

Note: PCA—principal component factor analysis; * same study sample; CALD—Cultural and Linguistic Diversity scale; CEF—Clinical Evaluation Form; CLE—Clinical Learning Environment scale; CLECS—Clinical Learning Environment Comparison Survey; CLEDI—Clinical Learning Environment Diagnostic Inventory; CLEI—Clinical Learning Environment Inventory; CLEI-19—Clinical Learning Environment Inventory 19 items; CLEQEI—Clinical Learning Environment Quality Evaluation Index; CLES—Clinical Learning Environment and Supervision Instrument; CLES-T—Clinical Learning Environment, Supervision, and Nurse Teacher; DREEM—Dundee Ready Education Environment Measure; EAPAP—Escala de Apoyo Académico en el Prácticum in Spanish; ESECS—Escala de Satisfação com as Experiências Clínicas Simuladas; SECEE—Student Evaluation of Clinical Education Environment.

**Table 2:** Evaluation of content validity and psychometric properties and development of recommendations for the development of the instruments.

Tool	Relevance	Comprehensiveness	Comprehensibility	Overall Content Validity	Structural Validity	Internal Consistency	Other Measurement	Recommendation
**CALD**	+/M	+/M	+/M	+/M	−/L	+/L	Hypothesis testing +/L Cross-cultural validity +/L	**A**
**CEF**	+/L	±/L	±/L	±/L		+/H		**B**
**CLE**	+/VL	±/VL	±/VL	±/VL	−/M	−/M		**C**
**CLECS**	+/M	±/M	±/M	±/M	−/L	+/L	Cross-cultural validity +/L Reliability -/L Hypothesis testing convergent +/L Hypothesis testing discriminant -/L	**B**
**CLEDI**	+/L	±/L	±/L	±/L	?/M	+/M	Criterion validity +/M Reliability +/M Hypothesis testing +/M	**B**
**CLEI**	+/M	±/M	±/M	±/M	?/VL	−/VL	Hypothesis testing +/VL	**C**
**CLEI-19**	+/M	±/M	±/M	±/M	+/H	+/H	Hypothesis testing +/H	**B**
**CLEQEI**	+/L	±/L	±/L	±/L	+/H	+/H	Reliability +/H Hypothesis testing +/H	**B**
**CLES**	±/M	±/M	±/M	±/M	?/L	+/L	Cross-cultural testing +/L Reliability +/L Hypothesis testing +/L	**B**
**CLES-T**	±/M	±/M	±/M	±/M	−/L	+/L	Reliability −/VL Hypothesis testing ?/VL Cross-cultural validity +/VL	**B**
**DREEM**	+/M	+/M	+/M	+/M	−/L	+/L	Hypothesis testing +/L Cross-cultural validity +/VL	**A**
**EAPAP**	+/VL	±/VL	±/VL	±/VL	+/H	+/H		**B**
**ESECS**	+/M	+/M	+/M	+/M	−/VL	−/VL		**B**
**SECEE**	+/M	+/M	+/M	+/M	?/H	+/H	Cross-cultural validity +/H Reliability −/H Hypothesis testing +/H	**A**

Note: +—sufficient; -—insufficient; ±—inconsistent; ?—indeterminate; H—High; M—Moderate; L—Low; VL—Very low; A—sufficient content validity (any level) and at least low quality evidence for sufficient internal consistency; B—non A and non C; C—high quality evidence for an insufficient measurement property; CALD—Cultural and Linguistic Diversity scale; CEF—Clinical Evaluation Form; CLE—Clinical Learning Environment scale; CLECS—Clinical Learning Environment Comparison Survey; CLEDI—Clinical Learning Environment Diagnostic Inventory; CLEI—Clinical Learning Environment Inventory; CLEI-19—Clinical Learning Environment Inventory 19 items; CLEQEI—Clinical Learning Environment Quality Evaluation Index; CLES—Clinical Learning Environment and Supervision Instrument; CLES-T—Clinical Learning Environment, Supervision, and Nurse Teacher; DREEM—Dundee Ready Education Environment Measure; EAPAP—Escala de Apoyo Académico en el Prácticum in Spanish; ESECS—Escala de Satisfação com as Experiências Clínicas Simuladas; SECEE—Student Evaluation of Clinical Education Environment.

**Table 3:** Categories associated with instruments.

Categories	Tools														
	CALD	CEF	CLE	CLECS	CLEDI	CLEI	CLEI-19	CLEQEI	CLES	CLES-T	DREEM	EAPAP	ESECS	SECEE	F
Learning the nursing process				X											1
Self-learning	X							X							2
Self-efficacy in practical learning				X				X							2
Self-efficacy in theoretical learning											X	X			2
Students’ motivation	X					X			X		X		X		5
Learning opportunities		X			X	X		X	X	X	X		X	X	9
Learning barriers	X		X		X	X									4
Quality of relationship with teachers											X	X			2
Quality of relationship with tutors		X		X		X	X		X	X			X	X	8
Quality of the clinical learning environment	X		X		X	X		X	X	X				X	8
Quality of the classroom learning environment											X				1
Quality of the teaching strategies											X	X			2
Quality of the tutoring strategies	X	X		X	X	X	X	X	X	X			X	X	11
Quality of relationship with Staff nurse	X	X	X		X					X					5
Quality of relationship with patients and relatives				X											1
Safety and quality of care			X	X	X	X		X	X	X				X	8
Satisfaction with the practical training experience			X	X		X	X	X	X	X					7
Satisfaction with theoretical learning											X	X			2
Academic support (access to resources)		X	X		X							X		X	5
Academic support (information received)		X			X										2
Academic support (student support)											X				1
Support from the staff nurse					X		X		X					X	4
Support from fellow students			X								X		X		3

Note: CALD—Cultural and Linguistic Diversity scale; CEF—Clinical Evaluation Form; CLE—Clinical Learning Environment scale; CLECS—Clinical Learning Environment Comparison Survey; CLEDI—Clinical Learning Environment Diagnostic Inventory; CLEI—Clinical Learning Environment Inventory; CLEI-19—Clinical Learning Environment Inventory 19 items; CLEQEI—Clinical Learning Environment Quality Evaluation Index; CLES—Clinical Learning Environment and Supervision Instrument; CLES-T—Clinical Learning Environment, Supervision, and Nurse Teacher; DREEM—Dundee Ready Education Environment Measure; EAPAP—Escala de Apoyo Académico en el Prácticum in Spanish; ESECS—Escala de Satisfação com as Experiências Clínicas Simuladas; SECEE—Student Evaluation of Clinical Education Environment; F—frequency of appearance of the category on scales.

## Data Availability

None.

## Appendix A

Multimedia Appendix 1: Searching filter of PubMed

- **Construct** (“clinical practice*” OR “clinical internship” OR “clinical nursing education” OR “clinical education” OR “education-nursing” OR “practice education” OR “practicum education” OR “hospital learning environment” OR “nurse education” OR “clinical learning environment” OR “learning environment” OR “clinical placement” OR “clinical teaching” OR “mentoring” OR “tutoring”)
- **Population** (“nurse student*” OR “baccalaureate student*” OR “student nurse*”)
- **Type of instruments** (instrument* OR tool* OR diar* OR scale* OR questionnaire* OR inventory)
- **Measurement properties (inclusion and exclusion filters):** ((instrumentation[sh] OR methods[sh] OR “Validation Studies”[pt] OR “Comparative Study”[pt] OR “psychometrics”[MeSH] OR psychometr*[tiab] OR clinimetr*[tw] OR clinometr*[tw] OR “outcome assessment (health care)”[MeSH] OR “outcome assessment”[tiab] OR “outcome measure*”[tw] OR “observer variation”[MeSH] OR “observer variation”[tiab] OR “reproducibility of results”[MeSH] OR reproducib*[tiab] OR “discriminant analysis”[MeSH] OR reliab*[tiab] OR unreliab*[tiab] OR valid*[tiab] OR “coefficient of variation”[tiab] OR coefficient[tiab] OR homogeneity[tiab] OR homogeneous[tiab] OR “internal consistency”[tiab] OR (cronbach*[tiab] AND (alpha[tiab] OR alphas[tiab])) OR (item[tiab] AND (correlation*[tiab] OR selection*[tiab] OR reduction*[tiab])) OR agreement[tw] OR precision[tw] OR imprecision[tw] OR “precise values”[tw] OR test-retest[tiab] OR (test[tiab] AND retest[tiab]) OR (reliab*[tiab] AND (test[tiab] OR retest[tiab])) OR stability[tiab] OR interrater[tiab] OR inter-rater[tiab] OR intrarater[tiab] OR intra-rater[tiab] OR intertester[tiab] OR inter-tester[tiab] OR intratester[tiab] OR intra-tester[tiab] OR interobserver[tiab] OR inter-observer[tiab] OR intraobserver[tiab] OR intra-observer[tiab] OR intertechnician[tiab] OR inter-technician[tiab] OR intratechnician[tiab] OR intra-technician[tiab] OR interexaminer[tiab] OR inter-examiner[tiab] OR intraexaminer[tiab] OR intra-examiner[tiab] OR interassay[tiab] OR inter-assay[tiab] OR intraassay[tiab] OR intra-assay[tiab] OR interindividual[tiab] OR inter-individual[tiab] OR intraindividual[tiab] OR intra-individual[tiab] OR interparticipant[tiab] OR inter-participant[tiab] OR intraparticipant[tiab] OR intra-participant[tiab] OR kappa[tiab] OR kappa’s[tiab] OR kappas[tiab] OR repeatab*[tw] OR ((replicab*[tw] OR repeated[tw]) AND (measure[tw] OR measures[tw] OR findings[tw] OR result[tw] OR results[tw] OR test[tw] OR tests[tw])) OR generaliza*[tiab] OR generalisa*[tiab] OR concordance[tiab] OR (intraclass[tiab] AND correlation*[tiab]) OR discriminative[tiab] OR “known group”[tiab] OR “factor analysis”[tiab] OR “factor analyses”[tiab] OR “factor structure”[tiab] OR “factor structures”[tiab] OR dimension*[tiab] OR subscale*[tiab] OR (multitrait[tiab] AND scaling[tiab] AND (analysis[tiab] OR analyses[tiab])) OR “item discriminant”[tiab] OR “interscale correlation*”[tiab] OR error[tiab] OR errors[tiab] OR “individual variability”[tiab] OR “interval variability”[tiab] OR “rate variability”[tiab] OR (variability[tiab] AND (analysis[tiab] OR values[tiab])) OR (uncertainty[tiab] AND (measurement[tiab] OR measuring[tiab])) OR “standard error of measurement”[tiab] OR sensitiv*[tiab] OR responsive*[tiab] OR (limit[tiab] AND detection[tiab]) OR “minimal detectable concentration”[tiab] OR interpretab*[tiab] OR ((minimal[tiab] OR minimally[tiab] OR clinical[tiab] OR clinically[tiab]) AND (important[tiab] OR significant[tiab] OR detectable[tiab]) AND (change[tiab] OR difference[tiab])) OR (small*[tiab] AND (real[tiab] OR detectable[tiab]) AND (change[tiab] OR difference[tiab])) OR “meaningful change”[tiab] OR “ceiling effect”[tiab] OR “floor effect”[tiab] OR “Item response model”[tiab] OR IRT[tiab] OR Rasch[tiab] OR “Differential item functioning”[tiab] OR DIF[tiab] OR “computer adaptive testing”[tiab] OR “item bank”[tiab] OR “cross-cultural equivalence”[tiab]))) NOT ((“addresses”[Publication Type] OR “biography”[Publication Type] OR “case reports”[Publication Type] OR “comment”[Publication Type] OR “directory”[Publication Type] OR “editorial”[Publication Type] OR “festschrift”[Publication Type] OR “interview”[Publication Type] OR “lectures”[Publication Type] OR “legal cases”[Publication Type] OR “legislation”[Publication Type] OR “letter”[Publication Type] OR “news”[Publication Type] OR “newspaper article”[Publication Type] OR “patient education handout”[Publication Type] OR “popular works”[Publication Type] OR “congresses”[Publication Type] OR “consensus development conference”[Publication Type] OR “consensus development conference, nih”[Publication Type] OR “practice guideline”[Publication Type]) NOT (“animals”[MeSH Terms] NOT “humans”[MeSH Terms]))
